# Is the Direct Fixation of Displaced Quadrilateral Plates in Acetabular Fractures Necessary?

**DOI:** 10.3390/jcm12226994

**Published:** 2023-11-09

**Authors:** Beom-Soo Kim, Ki-Cheor Bae, Chul-Hyun Cho, Kyung-Jae Lee, In Gyu Lee, Min-Gyu Lee, Byung-Woo Min

**Affiliations:** 1 Department of Orthopedic Surgery, Keimyung University Dongsan Hospital, Keimyung University School of Medicine, Daegu 42601, Republic of Korea; kbs090216@gmail.com (B.-S.K.); bkc@dsmc.or.kr (K.-C.B.); oscho5362@dsmc.or.kr (C.-H.C.); oslee@dsmc.or.kr (K.-J.L.); dlalsrb227@naver.com (M.-G.L.); 2 Marine Corps Education & Training Group, Republic of Korea Marine Corps, Pohang 37896, Republic of Korea; ingyu331@gmail.com

**Keywords:** acetabular fractures, quadrilateral plate, treatments, fixation, outcomes

## Abstract

Quadrilateral plate fractures represent a heterogeneous group of acetabular fractures. Accurate reduction is required to prevent post-traumatic arthritis. The purpose of this study is to determine the reduction effect of the direct fixation of quadrilateral plates in acetabular fractures, and to evaluate the strength of direct fixation compared to indirect fixation. Between 2005 and 2021, 49 patients underwent surgery for open reduction and internal fixation in acetabular fractures with severely displaced quadrilateral plates. Twenty-nine patients comprised the indirect fixation group, and twenty patients comprised the direct fixation group. In a comparison of primary outcome between two groups, 10 out of 29 indirect-group patients and 1 out of 20 direct-group patients developed post-traumatic osteoarthritis, wherein the difference between the two groups is statistically significant. In the assessment of postoperative Matta’s radiological reduction status, 19 out of 20 patients in the direct group had achieved anatomical and congruent reduction. The treatment using a direct reduction and internal fixation improved the reduction quality of articular displacement and offered a better survivorship of the affected hip joint.

## 1. Introduction

There are many challenges in the management of acetabular fractures and associated injuries [1,2,3]. As a result of complex surgical approaches and the difficulty of achieving an anatomical reduction in displaced fracture fragments, the surgical process is frustrating, with a steep learning curve and demanding patient care [4,5,6,7,8].

Since the types of acetabular fractures were classified by Judet and Letournel [9,10] and several important factors in the surgical treatment of acetabular fractures were identified, a quadrilateral plate has not been specifically considered as a measure in systems for the classification of acetabular fractures [11,12,13,14,15].

A quadrilateral plate fracture is indicated by the displacement of the acetabular medial wall [16]. The occurrence of these heterogeneous subtypes is increasing, along with an increasing number of patients with osteoporotic acetabular fractures [17,18,19].

Many methods for the management of quadrilateral plate fractures have been reported [20,21,22]. Some fractures can be fixed indirectly; however, the use of indirect fixation could cause difficulty in achieving a congruent, anatomical reduction in the hip joint in comminuted, multi-fragmentary fractures. Other studies described the direct reduction in the quadrilateral plate and fixation using various types of plates or implants [23].

There is no consensus with regard to whether or not the use of the direct fixation of a quadrilateral plate results in a better outcome for an acetabular fracture with a displaced quadrilateral plate. Therefore, the purpose of this study is to determine the reduction effect of direct fixation for quadrilateral plates in acetabular fractures, and to evaluate the strength of direct fixation compared to indirect fixation.

## 2. Materials and Methods

### 2.1. The Study Group

We retrospectively identified a consecutive series of sixty patients who underwent surgery for open reduction and internal fixation of acetabular fractures with displaced quadrilateral plates from November 2005 to February 2021. Adult patients who underwent surgery for the treatment of acetabular fractures with displaced quadrilateral plates were included. Patients with a follow-up period of more than one year were selected, and patients who were lost to follow-up during the follow-up period were excluded. This latter group of patients consisted of those lost to death during the period of follow-up after discharge, and a loss of contact addresses on record. Finally, 49 patients were enrolled in this study.

**Figure 1:**
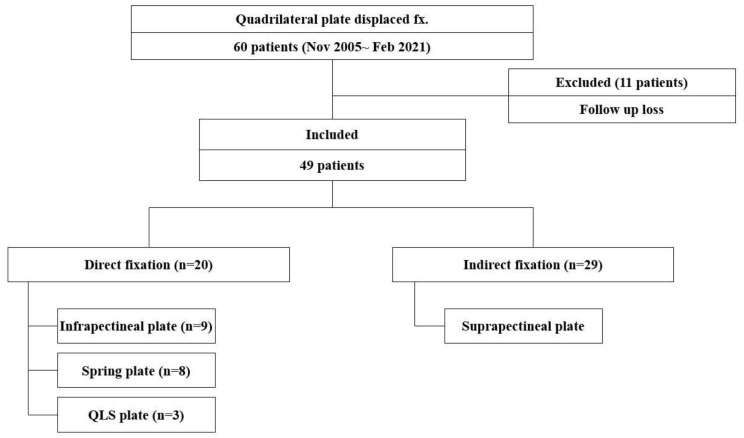
Patient’s flow chart. Patient profiles and the groups included in the study. QLS = quadrilateral surface.

Patients were divided into two groups; the Indirect group, who underwent surgery using indirect fixation using a suprapectineal plate only, and the Direct group, who underwent surgery using direct reduction and fixation using an infrapectineal plate or a spring plate or a quadrilateral surface (QLS) plate (Pro–Pelvis and Acetabulum System^®^, Stryker, Kalamazoo, MI, USA). The Indirect group included 29 cases, and the Direct group included 20 cases—eight cases with contoured spring plate fixation, nine cases with an infrapectineal plate, and three cases with QLS plate fixation (Figure 1).

### 2.2. Surgical Procedures

**Figure 2:**
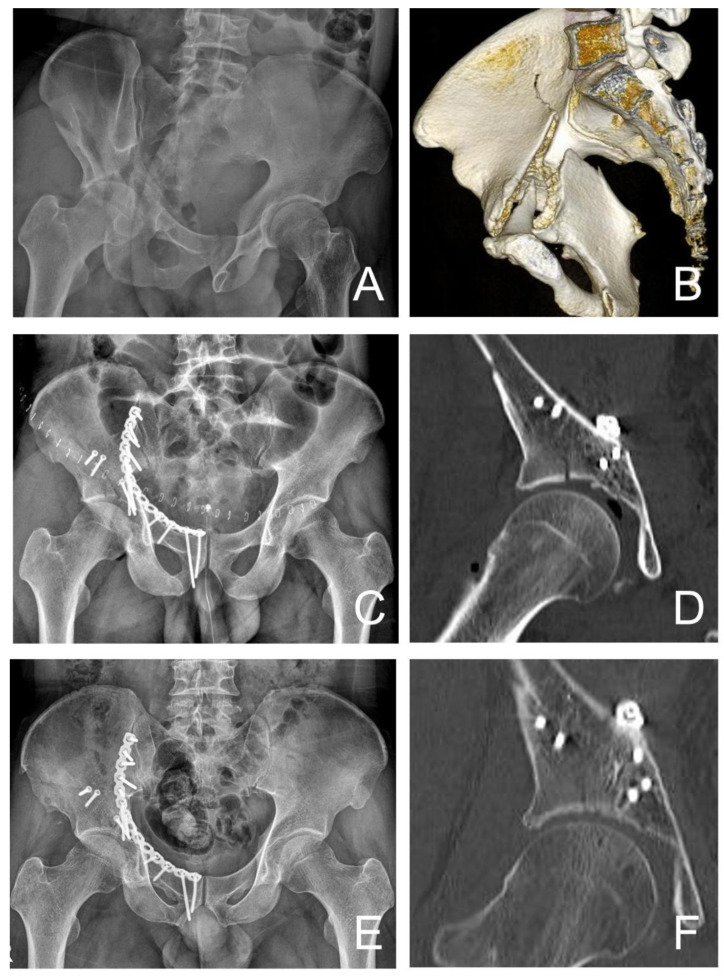
A 41-year-old man underwent indirect fixation with a suprapectineal plate. (**A**) Radiograph shows both-column fracture with 30 mm displacement. (**B**) Three-dimensional computed tomography (CT) scan shows both-column fracture with quadrilateral plate displacement. (**C**) Indirect fixation with suprapectineal plate is performed. (**D**) CT scan shows congruent reduction. (**E**) Radiograph obtained 41 months after surgery shows union at the fracture site and good grade with Matta’s outcome grading. (**F**) CT scan also shows mild joint space narrowing, but no sign of osteophyte, subchondral sclerosis, and femoral head collapse. CT = computed tomography.

In the chronological point of view, a conventional ilioinguinal approach for acetabular fractures with quadrilateral plate displacement was used before April 2014. That is the reason why there were only patients with indirectly fixed quadrilateral plates and relatively longer follow-up periods before April 2014. Use of the ilioinguinal approach creates three anatomical windows for exposure of the fracture site. First, the lateral window is placed between ilium and iliopsoas. Second, the middle window is located between the iliopsoas muscle and the iliac neurovascular bundle. Lastly, the third window, or medial window, is located between the neurovascular bundle and the inguinal spermatic cord or round ligament. The middle window allows an approach to the distal pelvic brim and the quadrilateral plate [24] (Figure 2).

**Figure 3:**
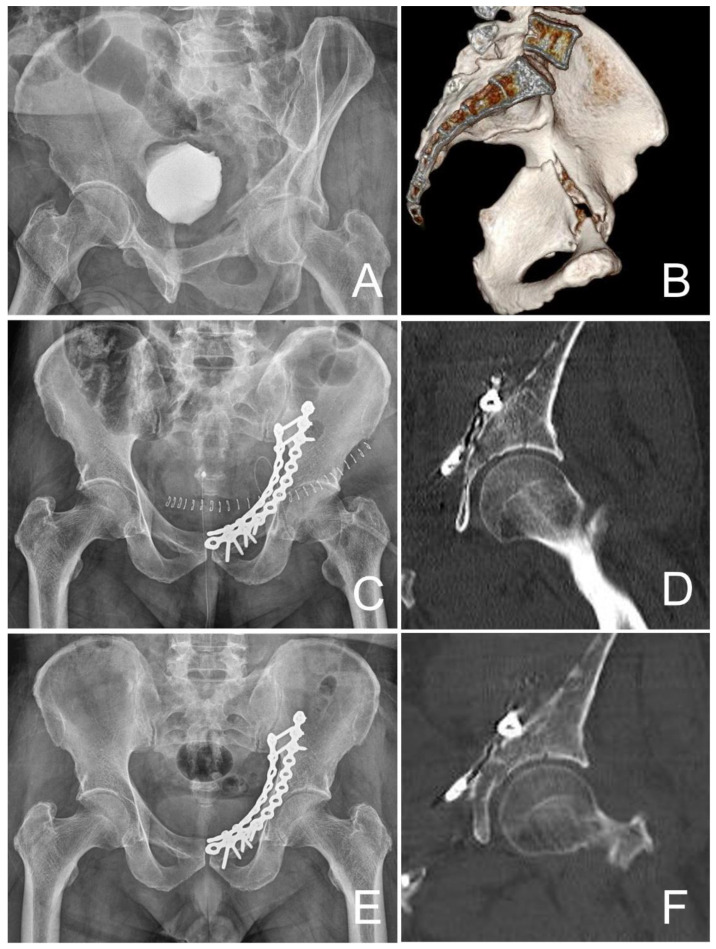
A 66-year-old man underwent direct fixation with infrapectineal plate. (**A**) Radiograph shows both-column fracture with 13 mm displacement. (**B**) Three-dimensional computed tomography (CT) scan shows both-column fracture with quadrilateral plate displacement. (**C**) Direct reduction and fixation with infrapectineal plate is performed. (**D**) CT scan shows anatomical reduction. (**E**) Radiograph obtained 24 months after surgery shows union at the fracture site and excellent grade with Matta’s outcome grading. (**F**) CT scan also shows no sign of osteophyte, joint space narrowing, subchondral sclerosis, and femoral head collapse. CT = computed tomography.

After 2014, the modified Stoppa approach was brought in for treatment of acetabular fractures. Publication of descriptions of using an infrapectineal plate through use of a modified Stoppa approach began after 2006 [25,26], and this approach has been used for full-scale clinical use for the management of acetabular fractures with displaced quadrilateral plate since 2014 (Figure 3). Using this approach, the reduced quadrilateral plate could be fixed directly during surgery.

### 2.3. Radiologic Evaluation

Acetabular fracture patterns were analyzed and sorted according to the Judet and Letournel classification system [9,10,27]; the displacement patterns of the quadrilateral plate were also analyzed. Post-traumatic osteoarthritis is like the radiological characteristics of general degenerative osteoarthritis in the lower extremity joint, and in this study, Matta’s grading was used for the hip joint [28]. Using Matta’s grading system for radiological outcome, the assessment of the primary outcome was based on the postoperative survivorship of the hip joint as post-traumatic osteoarthritis [29]. Several radiologic findings, including osteophyte, joint space narrowing, subchondral sclerosis, and other factors, including femoral head collapse, were used in the determination of grades. After the measurement of the radiologic findings mentioned above, grades were given as excellent, good, fair, and poor. Excellent and good grades were classified as the success group, whereas fair and poor grades as the failure group [11,29]. 

In addition, the postoperative fracture reduction status was compared using Matta’s score [11,29]. Up to 1 mm of displacement after reduction is graded as anatomical reduction, 2 to 3 mm of displacement as congruent, and above 3 mm of displacement as poor reduction. In addition, a comparison of the medialization of femoral heads between two groups was performed after the surgery.

### 2.4. Postoperative Management

Regular prophylactic intravenous antibiotics (first cephalosporin) were administered to all patients for six days after surgery. Surgical-site drainage tube insertions were maintained for one to three days and removed when the volume of the drain remained below 30 mL within 24 h. 

After awakening from anesthesia, rehabilitation for patients was started with non-weight-bearing bedside exercise for approximately four weeks after surgery. Assisted weight bearing ambulation exercise using a walker or crutches was started at 4–8 weeks after surgery. 

All patients underwent postoperative radiologic evaluation after the surgery, including pelvic X-rays and computed tomography (CT) with a reconstruction of three-dimensional images. Follow up X-ray evaluations were performed 1, 3, 6, and 12 months after surgery, and CT follow-up was performed 6 and 12 months after surgery.

### 2.5. Statistical Analysis

Statistical analysis was performed using SPSS statistics package program (ver. 25.0; IBM Co., Armonk, NY, USA). To analyze the differences in demographic parameters, chi-squared tests and Mann–Whitney tests were used [30]. Bivariate comparisons using chi-squared tests were conducted for categorical variables. Continuous variables of the 2 groups were analyzed using Student-*t* test. The level of significance for all statistical comparisons was set at *p* < 0.05.

## 3. Results

Forty-nine patients were included in the study—forty males and nine females—and the average age was 49.4 years old. In the Direct group, the mean interval to surgery from initial trauma was seven days, and the average follow-up period was 20.0 months. In the Indirect group, the mean interval to surgery from initial trauma was 8.9 days, and the average follow-up period was 63.3 months (Table 1).

Associated both-column fracture was the most common type of fracture in both groups. In the Direct group, 13 out of 20 patients were diagnosed as associated both-column fracture; the other cases included five anterior columns, one anterior column with posterior hemitransverse, and one T-shaped fracture. In the Indirect group, 24 out of 29 patients were diagnosed as associated both-column fractures. And the Indirect group included four patients with anterior column fractures and one anterior column with posterior hemitransverse. Six cases had an acetabular-dome-impacted fracture pattern. One case was in the Direct group, which consisted of 5% of the group. The other five cases were in the Indirect group, which consisted of 17.2% of the group. In the Direct group, the fracture pattern of quadrilateral plate (QLP) had 15 cases of comminuted QLP. The other three patients showed a simple QLP fracture pattern. In the Indirect group, 22 patients (75.9%) had a comminuted QLP fracture, and the other seven patients (24.1%) had simple QLP fractures (Table 2).

A significantly better survivorship of the hip joint, which was regarded as the primary outcome after the fracture, was observed in the Direct group. In the Direct group, 19 (95%) patients were included in the success group, while in the Indirect group, 19 (65.5%) patients were included in the success group (*p* < 0.05) (Table 3).

An evaluation of postoperative reduction status with the degree of displacement of the fracture site was performed after the surgery. Reduction status was graded as anatomical, which was less than 1 mm of displacement, congruent (satisfactory) for 2–3 mm of displacement, and poor (unsatisfactory) for more than 3 mm of displacement. In the Direct group, 12 (60%) cases were measured as anatomical reduction, and seven (35%) cases as congruent cases. In the Indirect group, 13 (44.8%) cases were measured as anatomical reduction, nine (31%) cases as congruent, and seven (24.1%) cases as poor. A higher level of medialization of the femoral head after surgery was observed in the Indirect group. The mean degree of medialization was 0.3 mm in Direct group (range: 0–3 mm), but 3.9 mm in the Indirect group (range: 0–6 mm) (*p* < 0.001) (Table 4).

**Figure 4:**
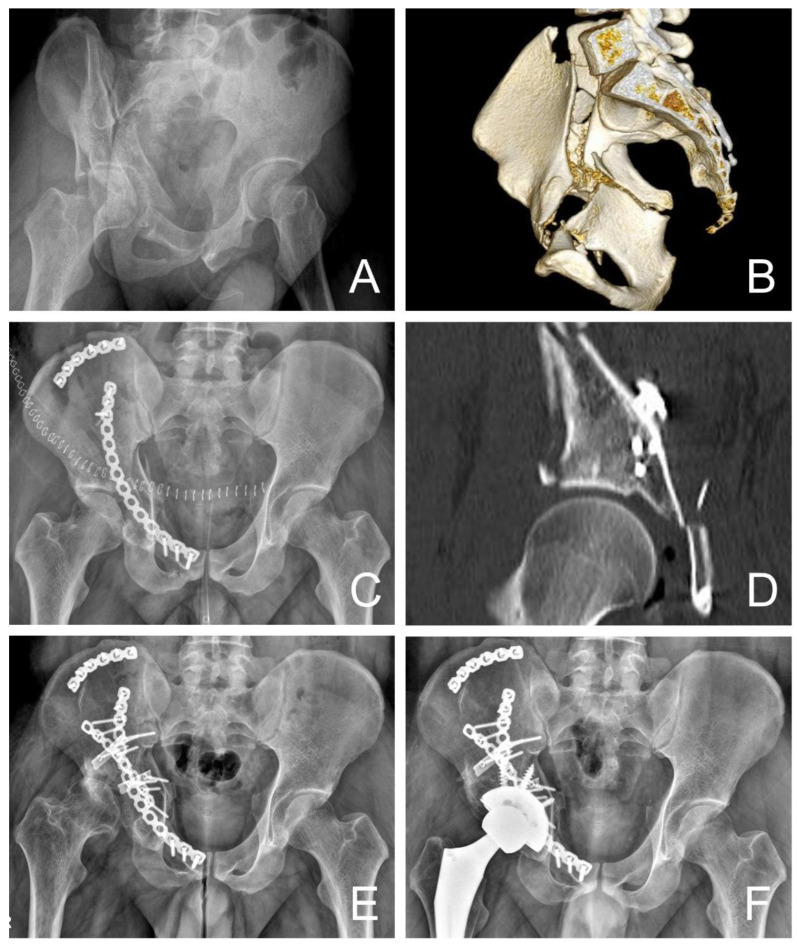
A 27-year-old man underwent indirect fixation with suprapectineal plate. (**A**) Radiograph shows both-column fracture with 20 mm displacement. (**B**) Three-dimensional computed tomography (CT) scan shows both-column fracture with quadrilateral plate displacement. (**C**) Indirect fixation with suprapectineal plate is performed. (**D**) CT scan shows poor reduction. (**E**) Radiograph obtained 17 months after surgery shows poor grade with Matta’s outcome grading with joint space narrowing above 50% and severe subchondral sclerosis. (**F**) Radiograph obtained after conversion of total hip arthroplasty, 17 months after initial surgery. CT = computed tomography.

Regarding postoperative complications, the major concerns were arthritis and hip joint survival. Patients who showed a development of arthritic change received either conservative management or conversion to total hip arthroplasty. Among the patients in this study, 1 out of 20 patients in the Direct group and 10 out of 29 patients in the Indirect group were considered as the failure group according to Matta’s grading system for radiologic outcome. Some of the patients are under conservative management, considering tolerable clinical symptoms; however, five of the patients were managed by converting to total hip arthroplasty (Figure 4).

## 4. Discussion

In this study, superior results were obtained from the use of the direct fixation group for the quadrilateral plate of acetabular fractures, not only for postoperative reduction status, but also for primary outcome regarding hip joint survival from arthritis based on radiologic evaluation. Therefore, the findings of this study might provide proof of the assumption that direct fixation of a quadrilateral plate fracture can result in better outcomes than with an indirect fixation of displaced quadrilateral plates.

The primary limitation of this study is the retrospective design of the research. Second, the number of patients in the groups are relatively small, particularly for the direct reduction group. Third, the timing of surgery for the two groups of patients was different, so the follow-up period was different. In addition, conducting future studies with a longer follow-up period and larger patient groups might be required in order to demonstrate the efficacy of the direct reduction in quadrilateral plate fractures compared to indirect reduction.

According to White et al. [20], there was previously a predominant trend toward conservative treatment; they emphasized the importance of operative treatment in order to achieve better outcomes for the patients. In their study, they compared the outcomes between patients who underwent surgical treatment after quadrilateral plate fractures with screws, pins, plates, and cerclage wiring or cables. They suggested that plating could currently be the most frequently employed method. However, although they compared the fixation method itself, they did not determine the exact effective value of whether the fractures are directly or indirectly reduced.

Karim et al. [21], who introduced the fixation method using a buttress screw, demonstrated some of the effect of the anatomical reduction in acetabular fractures with quadrilateral plates with fewer complications. They suggested the use of a plate with buttressing screw, which provides the advantage of avoiding the risk of hardware penetration to the joint, and the dissection of the quadrilateral plate is not required. Obviously, they suggested one method for reducing and fixing displaced quadrilateral plate fractures; however, when using this method to emphasize the efficacy of the buttressing screw, there is not much of a direct reduction.

Several factors can affect the prognosis after an operative treatment of acetabular fractures. An associated fracture or injury type, reduction status, the presence of joint dislocation, and the delay of surgery are known predictors of the outcome of surgically treated acetabular fractures [12,13,15]. Because a quadrilateral plate fracture is considered a heterogeneous subtype of acetabular fractures [20], failure to reconstruct the buttressing function of the medial wall can cause an incongruous hip and poor reduction status, resulting in worse outcome [31,32].

The conventional ilioinguinal approach for acetabular fractures has been widely used because of its advantage of allowing a wide view of some types of fracture patterns such as anterior and transverse fractures, with a low risk of vascular injury [33,34]. The use of a quadrilateral plate has also been manageable with the use of an ilioinguinal approach using implants such as a suprapectineal plate and buttressing screw [4,21]. However, when using this approach, because of complicated anatomic structures in this surgical field and steep learning curve, there is difficulty in identifying the exact fracture pattern and in obtaining an adequate operation view.

In an explanation of the modified Stoppa approach through the rectus abdominis muscle to reach the pelvic ring reported by Cole and Bolhofner [34], the main advantage was the infrapectineal plating and management of quadrilateral plate fractures because the use of this approach can allow for a direct assessment, not only to the posterior surface of the pubic ramus, pubic eminence, and infrapectineal surface, but also to the medial surface of acetabulum—in other words, the quadrilateral plate.

The purpose of this study is not to focus on the advantages of a certain surgical approach itself. In some way or another, the findings of this study demonstrated that the direct fixation of the displaced quadrilateral plate is superior to indirect fixation. Three methods were applied for directly fixing the quadrilateral plate: infrapectineal plate, spring plate, and QLS plate. Treatment with an infrapectineal plate was administered in eight patients. The reconstruction plates were over-bent and then attached to the infrapectineal surface, directly buttressing the quadrilateral plate and preventing secondary medialization of the femoral head. Treatment with a spring plate, which is a reconstruction plate bent over the pelvic brim buttressing the medial wall, was administered to the other eight patients. Treatment with a QLS plate was administered in the two remaining patients who underwent direct fixation. No significant difference in outcomes was observed among these fixation methods, which means that meaningful results can be obtained using direct fixation, no matter what method is used.

This study has several strengths that could support its results. First, the treatment of all patients included in this study was administered by a single surgeon at a single medical institution, which rules out other varieties of factors, which could alter not only the consistency of treatment protocols and evaluations, but also the result of the treatment. Second, the two patient treatment method groups (direct fixation, indirect fixation) with the same fracture patterns could be compared. No significant difference in the preoperatively evaluated fracture morphology and classification of fracture types was observed between the two groups of patients.

## 5. Conclusions

This study was designed to demonstrate the strength of the direct fixation of a displaced quadrilateral plate in acetabular fractures. This study included 49 patients who underwent treatment for acetabular fractures with a displaced quadrilateral plate between November 2005 and February 2021, presenting an analysis of several radiological findings to compare the result between direct fixation and indirect fixation of quadrilateral plates. The findings of this study demonstrate that the surgical treatment of acetabular fractures with displaced quadrilateral plates using direct fixation with buttress plates can improve the reduction quality of articular displacement and thus offer a better survivorship of the affected hip joint.

## Figures and Tables

**Table 1:** Demographic data.

	Direct Group (n = 20)	Indirect Group (n = 29)	Total (n = 49)	*p* Value
Index age	52.1	47.63	49.4	>0.05
Gender (male/female)	18:2	22:7	40:9	>0.05
Laterality (right/left)	10:10	15:14	25:24	>0.05
Interval from initial trauma to surgery (days)	7	8.9	8.1	>0.05
Follow-up period (months)	20.0	63.3	45.6	<0.05 *

* Statistically significant.

**Table 2:** Fracture morphology, pattern, and classification in both groups.

	Direct Group (n = 20)	Indirect Group (n = 29)	*p* Value
Fracture classification			
ABC	13	24	>0.05
Anterior column	5	4	>0.05
ACPHT	1	1	>0.05
T-shaped	1	0	>0.05
Transverse	0	0	>0.05
QLP fracture pattern			
Simple	5	7	>0.05
Comminuted	15	22	>0.05
Medial displacement (mm)	18.8	20.3	>0.05
Number of case with dome impaction	1	5	>0.05

ABC = associated both-column, QLP = quadrilateral plate, ACPHT = anterior column with posterior hemitransverse.

**Table 3:** Primary outcome according to Matta’s grading system for radiological outcome.

	Direct Group (n = 20)	Indirect Group (n = 29)	*p* Value
Success			<0.05 *
Excellent	12	16	
Good	7	3	
Failure			<0.05 *
Fair	0	4	
Poor	1	6	

* Statistically significant.

**Table 4:** Postoperative evaluations.

	Direct Group	Indirect Group	*p* Value
Postoperative reduction status			
Anatomical (<1 mm)	12	13	>0.05
Congruent (2–3 mm)	7	9	>0.05
Poor (>3 mm)	1	7	>0.05
Medialization of femoral head (mm)	0.3	3.9	<0.05 *

* Statistically significant.

## Data Availability

No new data were created or analyzed in this study. Data sharing is not applicable to this article.
